# A population-based study of incidence and patient survival of small cell carcinoma in the United States, 1992–2010

**DOI:** 10.1186/s12885-015-1188-y

**Published:** 2015-03-27

**Authors:** Graça M Dores, Osama Qubaiah, Ankur Mody, Bassam Ghabach, Susan S Devesa

**Affiliations:** 1 Oklahoma City Veterans Affairs Health Care System, Oklahoma City, OK 73104 USA; 2 Department of Health and Human Services, Division of Cancer Epidemiology and Genetics, National Cancer Institute, National Institutes of Health, Bethesda, MD 20892 USA; 3 Hematology and Oncology Associates, St. Louis, MO 63136 USA; 4 John Peter Smith Hospital, Fort Worth, TX 76104 USA; 5 University of North Texas Health Science Center, Fort Worth, TX 76106 USA

**Keywords:** Extrapulmonary small cell carcinoma, Small cell lung cancer, Epidemiology, Incidence, Survival

## Abstract

**Background:**

In contrast to the well-described epidemiology and behavior of small cell lung carcinoma (SCLC), little is known about extrapulmonary small cell carcinoma (EPSCC).

**Methods:**

Using data from the Surveillance, Epidemiology and End Results (SEER) Program (1992–2010), we calculated age-adjusted incidence rates (IRs), IR ratios (IRRs), annual percent change (APC), relative survival (RS), RS ratios (RSRs), and the respective 95% confidence intervals (95% CI) of SCLC and EPSCC according to primary site. We used the SEER historic stage variable that includes localized (confined to the organ of origin), regional (direct extension to adjacent organ/tissue or regional lymph nodes), and distant (discontinuous metastases) stages and combined localized and regional stages into “limited” stage.

**Results:**

The incidence of SCLC (IR = 76.3/million person-years; n = 51,959) was 22-times that of EPSCC (IR = 3.5; n = 2,438). Of the EPSCC sites, urinary bladder, prostate, and uterine cervix had the highest incidence (IRs = 0.7-0.8); urinary bladder (IRR = 4.91) and stomach (IRR = 3.46) had the greatest male/female disparities. Distant-to-limited stage site-specific IRRs of EPSCC were significantly elevated for pancreas (IRR = 6.87; P < 0.05), stomach, colon/rectum, ovary, and prostate (IRRs = 1.62-2.42; P < 0.05) and significantly decreased for salivary glands, female breast, uterine cervix, and urinary bladder (IRRs = 0.32-0.46). During 1992–2010, significant changes in IRs were observed for EPSCC overall (APC = 1.58), small cell carcinoma of the urinary bladder (APC = 6.75), SCLC (APC = −2.74) and small cell carcinoma of unknown primary site (APC = −4.34). Three-year RS was significantly more favorable for patients with EPSCC than SCLC for both limited (RSR = 2.06; 95% CI 1.88, 2.26) and distant stages (RSR = 1.55; 95% CI 1.16, 2.07). Among limited stage small cell carcinoma, RS was most favorable for salivary glands, female breast, and uterine cervix (RS = 52-68%), whereas RS for nearly all sites with distant stage disease was <10%.

**Conclusion:**

EPSCC comprises a heterogeneous group of diseases that appears, at least in part, etiologically distinct from SCLC and is associated with more favorable stage-specific patient survival.

**Electronic supplementary material:**

The online version of this article (doi:10.1186/s12885-015-1188-y) contains supplementary material, which is available to authorized users.

## Background

In the broad spectrum of neuroendocrine tumors, small cell carcinomas comprise the less differentiated tumors associated with aggressive behavior [1]. Most of what is known regarding the epidemiology of small cell carcinoma is derived from studies of lung cancer. Indeed, of the major lung cancer types, cigarette smoking has been most strongly associated with small cell lung carcinoma (SCLC) [2,3]. Risk factors for extrapulmonary small cell carcinoma (EPSCC) are unknown. While population-based epidemiologic studies of neuroendocrine tumors have considered incidence according to anatomic site, most have excluded small cell histology, based on the assumption that these aggressive, highly fatal tumors are etiologically distinct from their well-differentiated counterparts [4-7]. Few studies of EPSCC have assessed incidence and patient survival by site [8,9], and although some studies have focused on selected sites [10-12], to our knowledge, none have been comprehensive in their inclusion of topography while describing site-specific incidence rates and patient survival. To gain insight into the etiology and behavior of small cell carcinoma, we comprehensively assessed SCLC and EPSCC incidence and patient survival using population-based data from the National Cancer Institute’s Surveillance, Epidemiology and End Results (SEER) Program.

## Methods

We assessed incidence of small cell carcinoma based on cases diagnosed among residents of 13 cancer registry areas of the SEER (SEER-13) Program during 1992–2010. SEER-13 represents approximately 14% of the population of the U.S. and includes the states of Connecticut, Hawaii, Iowa, New Mexico, and Utah and the areas of Detroit, Michigan; San Francisco, Los Angeles, and San Jose-Monterey, California; Seattle-Puget Sound, Washington; Atlanta and rural Georgia; and the Alaska Native Tumor Registry. The SEER Program classifies histology and topography information according to the International Classification of Diseases for Oncology (ICD-O), and all cases have been recoded to the third edition of ICD-O (ICD-O-3) by the SEER Program [13].

Using SEER*Stat, version 8.1.2 (www.seer.cancer.gov), we calculated incidence rates (IRs), IR ratios (IRRs), and corresponding 95% confidence intervals (CIs) for all cases of microscopically confirmed small cell carcinoma (ICD-O-3 morphology codes 8041–8045) with malignant behavior (ICD-O-3 behavior code/3) according to primary site (topography codes specified in Table 1). Overall, 834 cases (1.5% of total) were not microscopically confirmed and were excluded from the study. Malignant tumor, small cell type (8002) was excluded due to being a nonspecific code and potentially including other malignancies characterized by small cells (e.g., malignant melanoma, lymphoma) [14]. All IRs were age-adjusted to the 2000 U.S. standard population and expressed per one million person-years (PY). IRs were assessed according to gender, age, calendar year, and stage. To allow a general overview of stage across primary sites, we used the SEER historic stage variable that includes localized (confined to the organ of origin), regional (direct extension to adjacent organ/tissue or regional lymph nodes), distant (discontinuous metastases), and unspecified stages. We combined localized and regional stages into the category of “limited” stage and maintained the distant stage variable as defined in SEER. Our “limited” and “distant” stage categories are intended to approximate the two-tier SCLC staging systems of the Veterans Administration Lung Study Group and the International Association for the Study of Lung Cancer [15].

**Table 1:** Age-adjusted incidence rates and incidence rate ratios of small cell lung carcinoma and extrapulmonary small cell carcinoma according to primary site and gender, SEER-13, 1992-2010*

	ICD-O-3	Males		Females		Male-to-female	
Site	topography codes	No.	IR	No.	IR	IRR	(95 % CI)
Total^§^	00.0-80.9	28,820	96.20	26,902	71.44	1.35	(1.32, 1.37)^†^
SCLC	34.0-34.9	26,798	89.19	25,161	66.88	1.33	(1.31, 1.36)^†^
EPSCC	00.0-33.9, 35.0-75.9, 77.0-77.9	1,272	4.41	1,166	3.06	1.44	(1.33, 1.56)^†^
Salivary glands	07.9-08.9	44	0.16	<16	~	~	
Esophagus	15.0-15.9	106	0.36	81	0.21	1.72	(1.27, 2.33)^†^
Stomach	16.0-16.9	64	0.21	24	0.06	3.46	(2.12, 5.80)^†^
Colon/rectum	18.0-20.9	125	0.42	113	0.30	1.41	(1.08,1.84)^†^
Anus	21.0-21.8	16	0.05	21	0.06	0.95	(0.46, 1.91)
Liver/intrahepatic bile ducts	22.0-22.1	18	0.06	<16	~	~	
Gallbladder	23.9	<16	~	30	0.08	~	
Pancreas	25.0-25.9	89	0.30	84	0.22	1.38	(1.01, 1.88)^†^
Nose, nasal cavity, middle ear	30.0-30.1, 31.0-31.9	21	0.07	16	0.04	1.56	(0.77, 3.21)
Larynx	32.0-32.9	37	0.11	27	0.07	1.57	(0.93, 2.68)
Breast	50.0-50.9	<16	~	68	0.18	~	
Vagina	52.9	NA	NA	37	0.10	~	
Cervix	53.0-53.9	NA	NA	260	0.69	~	
Uterus	54.0-55.9	NA	NA	56	0.15	~	
Ovary	56.9	NA	NA	111	0.29	~	
Prostate	61.9	206	0.73	NA	NA	~	
Kidney and renal pelvis	64.9, 65.9	22	0.07	<16	~	~	
Urinary bladder	67.0-67.9	411	1.48	118	0.30	4.91	(3.99, 6.09)^†^
Unknown primary	76.0-76.8, 80.9	750	2.61	575	1.50	1.73	(1.55, 1.94)^†^

Abbreviations: *CI* confidence interval, *EPSCC* extrapulmonary small cell carcinoma, *ICD-O-3* third edition of the International Classification of Diseases for Oncology*, NA* not applicable, *No*. number of cases, *SCLC* small cell lung carcinoma, *SEER-13* 13 cancer registry areas of the Surveillance, Epidemiology and End Results Program, *~* IRs and IRRs are not calculated for <16 cases.

*Incidence rates are age-adjusted to the 2000 U.S. standard population and expressed per 1,000,000 person-years. IRRs are based on unrounded rates.

^†^95 % CI excludes 1.00 (based on unrounded upper and lower CI), and IRR is significant (P < 0.05).

^§^Specified sites with 1–15 total cases not shown in the table (ICD-O-3 code): tongue (01.1-0.29), gum and other mouth (03.0-03.9, 05.0-05.9, 06.0-06.9), tonsil (09.0-09.9), oropharynx (10.0-10.9), hypopharynx (12.9, 13.0-13.9), other oral cavity and pharynx (14.0-14.8), small intestine (17.0-17.9), intestinal tract, unspecified (26.0-26.9), soft tissues, including heart (38.0, 47.0-47.9 49.0-49.9), retroperitoneum/peritoneum (48.0-48.8), vulva (51.0-51.9), other female genital (57.0-58.9), testis (62.0-62.9), other male genital (63.0-63.9), ureter (66.9), eye and orbit (69.0-69.9), thyroid (73.9), other endocrine (37.9, 74.0-74.9, 75.0-75.9), and lymph nodes (77.0-77.9). Sites with >15 cases, but with fewer than 16 among both, males and females, not specified in the table (No., IR, ICD-O-3 code): nasopharynx (No. = 23; IR = 0.03; 11.0-11.9), other biliary (No. = 19; IR = 0.03; 24.0-24.9), and trachea/mediastinum/other respiratory (No. = 25; IR = 0.04; 33.9, 38.1-39.9).

Age-specific IRs (<15, 15–24, 25–34, 35–44, 45–54, 55–64, 65–74, 75+ years) were calculated and depicted on a log-linear scale as previously described [16]. Annual percent change (APC) in incidence was calculated using the weighted least squares method. We used the Joinpoint Regression Program (version 4.1.1.3) to assess the best fit for trend data and allowed up to 3 Joinpoints (http://surveillance.cancer.gov/joinpoint). Following the SEER Program convention, IRs are not presented for fewer than 16 cases [17].

Relative survival (RS) for cases diagnosed during 1992–2010 and followed through 2011 was estimated using the actuarial method in the SEER*Stat Survival Session. RS is the ratio of the proportion of observed survivors in a cohort of patients to the proportion of expected survivors in a comparable cohort of the general population [18]. We estimated 3-year RS, RS ratios (RSRs), and 95% CIs overall, according to site, stage, gender, age, primary tumor size, and calendar year. To allow comparison with previously published studies describing 5-year survival, we calculated 5-year RS for SCLC and EPSCC (Additional file 1: Table S1 and Additional file 2: Table S2). We excluded individuals with second or later primary cancers (n = 9,848), cases diagnosed by death certificate or autopsy (n = 134), those with unknown age (n = 4), and those alive with unknown survival time (n = 6). In total, 45,747 cases were available for the survival analysis. Following SEER convention, RS rates based on fewer than 25 cases are not presented [17].

## Results

Overall, 55,722 cases of small cell carcinoma were diagnosed among residents of SEER-13 during 1992–2010 (IR = 81.8/million PY). The incidence of SCLC (n = 51,959; IR = 76.3) was 22 times that of EPSCC (n = 2,438; IR = 3.5), accounting for 93% of cases of small cell carcinoma. Of the extrapulmonary sites, IRs were highest for urinary bladder, prostate, and uterine cervix (Table 1). Small cell carcinoma IR was 35% higher among men than women, with the greatest gender disparities noted for urinary bladder and stomach (male-to-female (M/F) IRR = 4.91 and 3.46, respectively).

**Figure 1:**
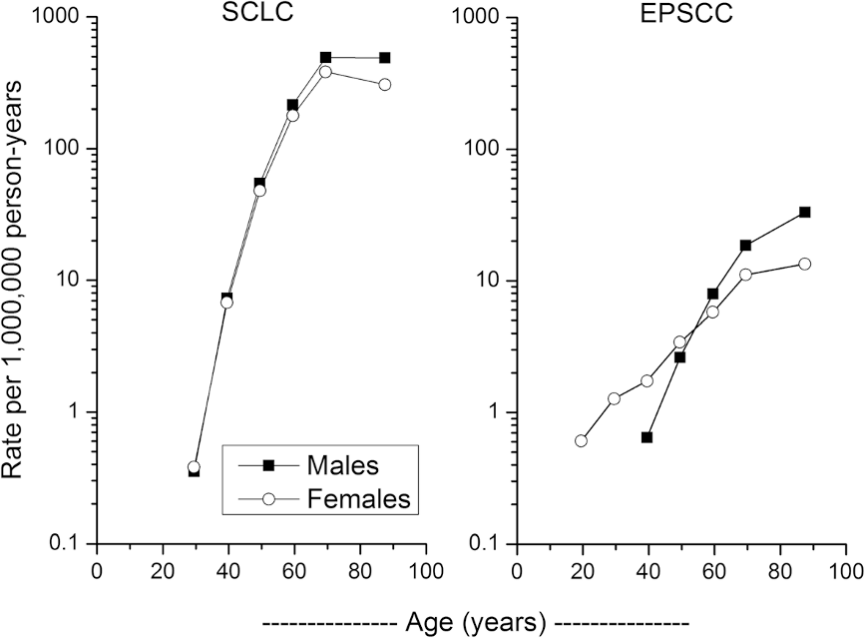
**Age-specific incidence rates of small cell lung carcinoma and extrapulmonary small cell carcinoma diagnosed in 13 cancer registry areas of the Surveillance, Epidemiology and End Results Program during 1992–2010 according to gender.**

IRs of SCLC and EPSCC increased exponentially with advancing age among men and women, most rapidly for SCLC among both men and women and least rapidly for EPSCC among women (Figure 1). Significant gender differences in SCLC IRs were apparent beginning at ages 45–54 years (M/F IRR = 1.14; 95% CI 1.07, 1.20), peaking at the oldest age group (≥75 years; M/F IRR = 1.60; 95% CI 1.55, 1.65). In contrast, the M/F IRRs for EPSCC rose from a female excess through ages 45–54 years (M/F IRR = 0.77; 95% CI 0.60, 0.97) to a male excess starting at ages 55–64 years (M/F IRR = 1.38; 95% CI 1.14, 1.67) and increasing progressively until ≥75 years (IRR = 2.47; 95% CI 2.14, 2.85). Among EPSCC diagnosed prior to age 55 years, uterine cervix (n = 156) and ovary (n = 75) comprised 61% of 379 cases among women, whereas the urinary bladder (n = 37) and colon/rectum (n = 33) accounted for the largest proportion (39%) of the 179 cases among men.

In contrast to SCLC where incidence of distant stage disease predominated over limited stage (distant/limited IRR = 2.32, 95% CI 2.28, 2.37), incidence of limited stage EPSCC was significantly higher than distant stage (distant/limited IRR = 0.89, 95% CI 0.82, 0.97) (Table 2). The distant/limited stage IRR was significantly elevated for small cell carcinoma of the stomach, colon/rectum, pancreas, ovary, and prostate, whereas IRRs were significantly decreased for salivary glands, female breast, uterine cervix, and urinary bladder.

**Table 2:** Age-adjusted incidence rates and incidence rate ratios of small cell lung carcinoma and extrapulmonary small cell carcinoma according to primary site and stage, SEER-13, 1992-2010*

	Limited		Distant		Not specified		Distant-to-limited	
Site	No.	IR	No.	IR	No.	IR	IRR	(95%CI)
Total	16,142	23.69	35,813	52.51	3,767	5.57	2.22	(2.18, 2.26)^†^
SCLC	14,963	21.98	34,770	50.99	2,226	3.30	2.32	(2.28, 2.37)^†^
EPSCC	1,179	1.71	1,043	1.52	216	0.31	0.89	(0.82, 0.97)^†^
Salivary glands	37	0.06	16	0.02	<16	~	0.44	(0.23, 0.81)^†^
Esophagus	73	0.11	93	0.14	21	0.03	1.27	(0.92, 1.75)
Stomach	22	0.03	53	0.08	<16	~	2.42	(1.45, 4.17)^†^
Colon/rectum	76	0.11	152	0.22	<16	~	1.95	(1.47, 2.61)^†^
Gallbladder	16	0.02	24	0.04	<16	~	1.48	(0.75, 2.97)
Pancreas	20	0.03	140	0.21	<16	~	6.87	(4.30, 11.61)^†^
Breast (female)	47	0.12	21	0.05	<16	~	0.44	(0.25, 0.75)^†^
Cervix	174	0.46	80	0.21	<16	~	0.46	(0.35, 0.60)^†^
Uterus	28	0.07	25	0.07	<16	~	0.91	(0.51, 1.62)
Ovary	35	0.09	72	0.19	<16	~	2.11	(1.38, 3.25)^†^
Prostate	66	0.23	108	0.38	32	0.11	1.62	(1.18, 2.25)^†^
Urinary bladder	390	0.58	123	0.18	16	0.02	0.32	(0.26, 0.39)^†^

Abbreviations: *CI* confidence interval, *EPSCC* extrapulmonary small cell carcinoma, *No.* number of cases, *SCLC* small cell lung carcinoma, *SEER-13* 13 cancer registry areas of the Surveillance, Epidemiology and End Results (SEER) Program, *~* IRs are not calculated for <16 cases.

*Incidence rates are age-adjusted to the 2000 U.S. standard population and expressed per 1,000,000 person-years. IRRs are based on unrounded rates. To allow a general overview of stage across primary sites, we used the SEER historic stage variable that includes localized (confined to the organ of origin), regional (direct extension to adjacent organ/tissue or regional lymph nodes), distant (discontinuous metastases), and unspecified stages. We combined localized and regional stages into the category of “limited” stage and maintained the distant stage variable as defined in the SEER Program.

^†^95 % CI excludes 1.00 (based on unrounded upper and lower CI), and IRR is significant (P < 0.05).

**Figure 2:**
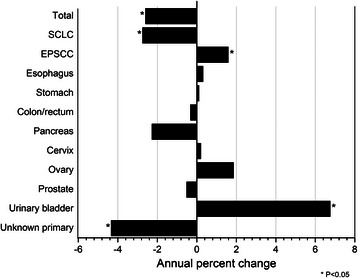
**Annual percent change of small cell lung carcinoma and extrapulmonary small cell carcinoma diagnosed in 13 cancer registry areas of the Surveillance, Epidemiology and End Results Program during 1992–2010 according to site.**

Whereas the incidence of SCLC decreased during 1992–2010 (APC = −2.74; P < 0.05), the incidence of EPSCC increased significantly (APC = 1.58; P < 0.05), largely related to the marked rise in small cell carcinoma of the urinary bladder (APC = 6.75; P < 0.05) (Figure 2). Similar to SCLC, only the incidence of small cell carcinoma of unknown primary site decreased over this timeframe (APC = −4.34; P < 0.05). APC did not change significantly for any other site for which APC could be calculated. All trend data were best fitted with 0 joinpoints.

Among patients with limited stage disease, 3-year RS was significantly more favorable for patients with EPSCC than SCLC overall, among males and females <60 and ≥60 years, whites <60 years, whites and blacks ≥60 years, according to tumor size, and by calendar year period (Table 3). Among patients with distant stage small cell carcinoma, RS was poor but signifcantly better for EPSCC than for SCLC overall, among white males <60 years of age, for tumor size >7 cm, and for cases diagnosed 2001–2010. Compared to 1992–2000, survival during 2001–2010 increased significantly for limited (limited/distant RSR = 1.22, 95% CI 1.13, 1.31) and distant stage (limited/distant RSR = 1.26, 95% CI 1.09, 1.45) SCLC but not for limited (limited/distant RSR = 1.07, 95% CI 0.90, 1.27) or distant stage (limited/distant RSR = 1.22, 95% CI 0.68, 2.18) EPSCC. To allow comparison of RS by EPSCC sites, we used uterine cervix as the referent site, since there were sufficient cases to allow stable comparisons for both, limited and distant stage disease (Table 4). Compared to small cell carcinoma of the uterine cervix, 3-year RS was significantly less favorable for limited stage small cell carcinoma of the esophagus (RSR = 0.64, 95% CI 0.42, 0.98) and urinary bladder (RSR = 0.78, 95% CI 0.62, 0.99), whereas for distant stage disease, pancreas was associated with significantly less favorable survival (RSR = 0.19, 95% CI 0.04, 0.96).

**Table 3:** Stage-specific three-year relative survival of patients with small cell lung carcinoma and extrapulmonary small cell carcinoma diagnosed in SEER-13 according to gender, age, and calendar year, 1992-2010*

	SCLC			EPSCC			EPSCC-to-SCLC	
Stage	No.	RS (%)	(95%CI)	No.	RS (%)	(95%CI)	RSR	(95%CI)
**Total** ^**†**^								
Limited	12,070	20.7	(19.9, 21.5)	907	42.7	(39.1, 46.2)	2.06	(1.88, 2.26)‡
Distant	29,003	4.0	(3.7, 4.2)	835	6.2	(4.6, 8.1)	1.55	(1.16, 2.07)‡
**Gender and age**								
Males, <60 years								
Limited	1,407	24.9	(22.6, 27.3)	124	37.0	(28.2, 45.7)	1.49	(1.15, 1.92)‡
Distant	3,993	4.8	(4.2, 5.5)	121	7.8	(3.8, 13.6)	1.63	(0.85, 3.11)
Females, <60 years								
Limited	1,442	31.2	(28.7, 33.7)	251	51.8	(45.2, 58.1)	1.66	(1.43, 1.93)‡
Distant	3,043	7.2	(6.2, 8.1)	169	11.1	(6.7, 16.8)	1.54	(0.96, 2.49)
Males, ≥60 years								
Limited	4,469	16.8	(15.6, 18.0)	312	40.3	(34.0, 46.6)	2.40	(2.02, 2.85)‡
Distant	11,594	2.8	(2.5, 3.1)	297	3.7	(1.8, 6.7)	1.32	(0.69, 2.53)
Females, ≥60 years								
Limited	4,752	19.7	(18.5, 21.0)	220	38.8	(31.6, 45.9)	1.97	(1.62, 2.40)‡
Distant	10,373	4.0	(3.6, 4.4)	248	4.9	(2.6, 8.4)	1.23	(0.67, 2.25)
**Race and age**								
Whites, <60 years								
Limited	2,360	28.9	(27.1, 30.8)	282	47.0	(40.8, 52.9)	1.63	(1.41, 1.88)‡
Distant	5,849	5.7	(5.1, 6.4)	224	10.1	(6.5, 14.7)	1.77	(1.16, 2.70)‡
Blacks, <60 years								
Limited	327	21.3	(16.9, 26.1)	44	25.1	(13.3, 38.9)	1.18	(0.67, 2.08)
Distant	796	5.1	(3.7, 6.9)	36	10.7	(3.2, 23.5)	2.10	(0.76, 5.81)
Whites, ≥60 years								
Limited	7,887	18.5	(17.6, 19.4)	449	38.7	(33.4, 43.8)	2.09	(1.81, 2.42)‡
Distant	18,793	3.3	(3.0, 3.6)	451	4.5	(2.7, 7.0)	1.36	(0.84, 2.21)
Blacks, ≥60 years								
Limited	699	16.5	(13.6, 19.6)	41	47.5	(30.6, 62.6)	2.88	(1.95, 4.25)‡
Distant	1,770	3.2	(2.4, 4.2)	50	4.3	(0.8, 13.1)	1.34	(0.33, 5.46)
**Primary tumor size**								
≤3 cm								
Limited	2,930	29.2	(27.5, 31.0)	187	57.1	(48.8, 64.6)	1.96	(1.68, 2.27)‡
Distant	4,469	5.5	(4.8, 6.2)	67	11.3	(4.7, 20.9)	2.05	(0.98, 4.32)
>3 cm - ≤7 cm								
Limited	3,391	21.6	(20.1, 23.1)	255	38.9	(32.3, 45.3)	1.80	(1.50, 2.16)‡
Distant	6,817	4.2	(3.7, 4.7)	210	3.7	(1.6, 7.0)	0.88	(0.41, 1.87)
>7 cm								
Limited	1,071	15.6	(13.3, 18.0)	117	37.0	(27.8, 46.3)	2.37	(1.76, 3.19)‡
Distant	3,079	4.9	(4.1, 5.7)	135	12.6	(7.3, 19.5)	2.57	(1.55, 4.27)‡
**Year of diagnosis**								
1992-2000								
Limited	6,660	18.9	(17.9, 19.9)	364	40.9	(35.5, 46.3)	2.16	(1.87, 2.50)‡
Distant	14,006	3.5	(3.2, 3.8)	314	5.5	(3.2, 8.5)	1.57	(0.98, 2.53)
2001-2010								
Limited	5,410	23.0	(21.7, 24.2)	543	43.8	(39.1, 48.4)	1.90	(1.69, 2.14)‡
Distant	14,997	4.4	(4.1, 4.8)	521	6.7	(4.6, 9.3)	1.52	(1.06, 2.19)‡

Abbreviations: *CI* confidence interval, *EPSCC* extrapulmonary small cell carcinoma, *No.* number, *RS* relative survival, *RSR* RS ratio, *SCLC* small cell lung carcinoma, *SEER-13* 13 cancer registry areas of the Surveillance, Epidemiology and End Results (SEER) Program.

* Based on microscopically confirmed cases of small cell carcinoma diagnosed during 1992–2010 and followed through 2011. To allow a general overview of stage across primary sites, we used the SEER historic stage variable that includes localized (confined to the organ of origin), regional (direct extension to adjacent organ/tissue or regional lymph nodes), distant (discontinuous metastases), and unspecified stages. We combined localized and regional stages into the category of “limited” stage and maintained the distant stage variable as defined in the SEER Program.

^†^Stage was not specified for 1,760 cases of SCLC (3-year RS (%) = 12.1, 95 % CI = 10.5, 13.8), and 162 cases of EPSCC (3-year RS (%) = 21.0, 95 % CI = 14.6, 28.2).

‡ 95 % CI excludes 1.00 (based on unrounded upper and lower CI), and RSR is significant (P < 0.05).

**Table 4:** Stage-specific three-year relative survival of patients with extrapulmonary small cell carcinoma diagnosed in SEER-13 according to site, 1992-2010*

	No.	RS (%)	(95%CI)	RSR	(95%CI)
**Limited stage**					
Uterine cervix	164	51.6	(43.3, 59.2)	1.00	
Salivary glands	27	67.7	(41.9, 83.9)	1.31	(0.93, 1.86)
Esophagus	56	33.2	(20.6, 46.3)	0.64	(0.42, 0.98)^†^
Stomach	18	~		~	
Colon/rectum	59	40.0	(26.5, 53.2)	0.78	(0.53, 1.13)
Pancreas	15	~		~	
Larynx	31	34.0	(17.7, 51.0)	0.66	(0.39, 1.12)
Female breast	39	62.5	(42.3, 77.3)	1.21	(0.88, 1.68)
Ovary	33	41.0	(23.9, 57.4)	0.79	(0.51, 1.24)
Prostate	50	36.4	(21.5, 51.4)	0.71	(0.45, 1.10)
Urinary bladder	256	40.5	(33.6, 47.4)	0.78	(0.62, 0.99)^†^
**Distant stage**					
Uterine cervix	74	9.4	(3.8, 18.0)	1.00	
Salivary glands	12	~		~	
Esophagus	75	1.4	(0.1, 6.7)	0.15	(0.02, 1.22)
Stomach	42	5.1	(0.9, 15.2)	0.54	(0.12, 2.56)
Colon/rectum	116	2.2	(0.4, 6.6)	0.23	(0.05, 1.10)
Pancreas	118	1.8	(0.3, 5.8)	0.19	(0.04, 0.96)†
Larynx	2	~		~	
Female breast	14	~		~	
Ovary	65	19.9	(10.8, 31.0)	2.12	(0.83, 5.37)
Prostate	88	7.4	(2.9, 14.9)	0.79	(0.26, 2.43)
Urinary bladder	87	2.9	(0.6, 9.0)	0.31	(0.07, 1.46)

Abbreviations: *CI* confidence interval, *No.* number, *RS* relative survival, *RSR* RS ratio, *SEER-13* 13 cancer registry areas of the Surveillance, Epidemiology and End Results Program, *~* relative survival not calculated for < 25 cases.

*Based on microscopically confirmed cases of small cell carcinoma diagnosed during 1992–2010 and followed through 2011. To allow a general overview of stage across primary sites, we used the SEER historic stage variable that includes localized (confined to the organ of origin), regional (direct extension to adjacent organ/tissue or regional lymph nodes), distant (discontinuous metastases), and unspecified stages. We combined localized and regional stages into the category of “limited” stage and maintained the distant stage variable as defined in the SEER Program.

^†^95 % CI excludes 1.00 (based on unrounded upper and lower CI), and RSR is significant (P < 0.05).

## Discussion

This is among the first population-based studies to describe distinct differences in incidence patterns between SCLC and EPSCC, suggesting etiologic differences, with the most convincing evidence arising from opposing temporal trends across sites. With the decrease in SCLC attributed to declining cigarette smoking, our findings raise the possibility that tobacco may have a less important role in the etiology of EPSCC overall, and also in small cell carcinoma of the bladder. Etiologic heterogeneity is also suggested by site-specific differences in incidence of EPSCC by gender, possibly reflecting varying environmental exposures and/or inherent susceptibility. Differences in stage at presentation of site-specific EPSCC may be due to distinct disease biology, since sites for which screening is available did not all present with less advanced disease (e.g., prostate), although diagnostic challenges could also affect stage at presentation. RS differences by site also suggest distinct biologic behavior and/or responsiveness to therapy.

Our findings differ from a 1970–2004 population-based study of 1,618 cases of EPSCC from South East England where EPSCC predominated among women (male:female case ratio of 1:1.3, comparable to a case ratio of 0.77) [9], in contrast to our case ratio of 1.09. In South East England, small cell carcinoma of the esophagus comprised the majority of EPSCC (18%), followed by stomach (6%) and prostate (6%). Among our 2,438 cases of EPSCC, the largest fractions were of the urinary bladder (22%), uterine cervix (11%), and colon/rectum (10%). While these findings may reflect differences in study design, calendar years of study, histologic entities included, or population characteristics, they also support potential differences in exposures or susceptibility between individuals in the U.S. and South East England. A literature review including more than 130 reports of gastrointestinal small cell carcinoma during 1970–2003 also identified esophagus as the most commonly reported primary site, accounting for 53% (n = 290/544 cases) of gastrointestinal small cell carcinomas [19]. While tobacco and alcohol use were found to be prevalent among patients in these series, an association with these or other putative risk factors has not been identified [19,20]. The differences in frequency of site-specific EPSCC across studies may reflect various factors, including time periods of study, accuracy of cancer reporting to cancer registries, varying extent of screening, distinct exposures among populations, diverse population characteristics (e.g., race/ethnicity), access to health care, and publication bias.

Among EPSCC, we report the highest incidence for the urinary bladder, a site that may clinically manifest early with hematuria or urinary symptoms, as supported by the more than triple number of cases diagnosed with limited stage than distant stage disease. The IRs were next highest for prostate and uterine cervix, both sites for which some form of cancer screening was available during the entire study period. For EPSCC of the cervix and female breast, there were more than twice as many cases with limited than distant stage, as would be expected in screen-detected cancers. However, there were 62% more distant than limited stage cases for the prostate, confirming findings in a prior SEER-based study (1973–2003) [21]. In combination, these findings raise the possibility that small cell carcinoma of the prostate may be associated with more aggressive biology than other sites for which screening is similarly available. However, alternate explanations, including a delay in diagnosis due to urinary symptoms being attributed to other causes, a missed finding of co-existing small cell carcinoma with adenocarcinoma of the prostate, or absence of elevation in prostate-specific antigen [22] could also account for the predominance of distant stage disease.

Incidence rates for SCLC and nearly all evaluable site-specific EPSCC were higher among males than females. This gender disparity in incidence has been similarly described for many other cancers [23]. While we noted a female predominance of EPSCC prior to age 55 years, this was driven by sex-specific cancer sites (uterine cervix, ovary). An early-onset incidence pattern has been described for cervical [24] and ovarian cancers [25], and whether human papillomavirus and hormonal factors, respectively, are risk factors for small cell carcinoma of these sites remains to be determined.

Lung cancer incidence rates among males and females have correlated with prior prevalence of tobacco use, in particular for SCLC and squamous cell carcinoma [26-28], thereby supporting the hypothesis that small cell carcinomas may share risk factors with non-small cell carcinomas occurring at the same site. Our study extends previous SEER-based reports [26,29], and we describe a continued decline in incidence of SCLC through 2010. In contrast to the significant decline in incidence during 1992–2010 observed for SCLC, a smoking-related cancer, the overall incidence of EPSCC increased. A rise in incidence was most notable for small cell carcinoma of the urinary bladder, despite cigarette smoking being an established risk factor for both lung and urinary bladder cancers. The increase in small cell carcinoma of the urinary bladder suggests a role for risk factor(s) other than tobacco, including occupational exposures. This finding is further supported by the decrease in incidence of papillary, squamous, and adenocarcinomas of the bladder since the early 1990s in the U.S., in contrast to the rise in small cell carcinoma previously described [30]. Therefore, the opposing trends of bladder cancer by histologic subtype makes early detection an unlikely explanation for the rising incidence of small cell carcinoma of the urinary bladder, as a similar direction in trend would be expected across histologic subtypes.

Consistent with some [8,9], but not all [31] prior reports, we found that RS was significantly more favorable for EPSCC than SCLC. In the U.S. and England, small cell carcinoma of female breast is associated with among the most favorable survival [8,9]. We also found survival for limited stage small cell carcinoma of salivary gland to be favorable, although based on few cases. Younger age and smaller tumor size were also associated with more favorable survival among limited stage SCLC and EPSCC. These findings are in agreement with a SEER-based study of EPSCC (1973–2006) where age ≥50 years, tumor size ≥5 cm, regional stage, and distant stage were identified as predictors of survival in multivariate analysis [8]. While several population-based studies [8,9,32] and single institution studies [31,33-41] have evaluated survival of EPSCC, comparison between studies is difficult due to varying measures of survival calculated, in addition to the extent to which staging and treatment information is considered; access to medical care is available; and distinct characteristics (e.g., race/ethnicity, socioeconomic status) are reflected in study populations. Additionally, with site-specific variation in survival of EPSCC, the entities included within the category of EPSCC across studies are likely to influence overall survival estimates.

A modest improvement in survival of SCLC has been reported since the 1970s and 1980s [42,43], and we observed a slight, but statistically significant, improvement in limited and extensive stage SCLC RS and a nonsignificant improvement in EPSCC RS subsequent to the 1990s. Despite statistical associations, clinically, the minimal change in survival over time likely reflects the lack of new therapies available for SCLC, with platinum agents remaining the mainstay of therapy since the 1980s [43]. Although the optimal treatment for EPSCC is unknown, it is often managed like SCLC [31], and while identification of new agents in the future may affect survival of both SCLC and EPSCC, variable response by site of disease might be expected based on historically reported differences in site-specific survival.

The strength of our population-based study includes the large size which allowed evaluation of incidence and patient survival by site. Despite its large size, we did not have sufficient cases of EPSCC to assess age-specific IRs, temporal trends, or RS for every specified site. Pathology was not centrally reviewed, so we cannot exclude the possibility of misclassification of other histologic entities characterized by small cells [14], including well differentiated neuroendocrine tumors. Our survival analyses did not include information on prognostic indicators such as performance status, lactate dehydrogenase, or weight loss because this information is not collected by the SEER Program. Additionally, we did not consider treatment or response to treatment in our survival analyses because treatment data (surgery, radiation) are limited to the first course of therapy, and information on chemotherapy, the mainstay of treatment for small cell carcinoma, is not publicly available. Lastly, our staging dichotomy (limited vs. distant stage) may have resulted in misclassification by stage, thereby yielding conservative RS estimates for limited stage disease and optimistic RS estimates for distant stage disease.

## Conclusions

In summary, distinct incidence patterns suggest that there are etiologic differences between SCLC and EPSCC. Opposing temporal trends for SCLC and EPSCC since the 1990s support a less important role for cigarette smoking in EPSCC overall than in SCLC. Gender disparities in incidence of site-specific EPSCC further implicate distinct exposures and/or inherent susceptibility differences by site. Disease biology of EPSCC also appears to differ by primary site, as demonstrated by some screen-detectable cancer sites presenting predominantly with limited stage disease (e.g., uterine cervix, female breast) in contrast to other sites where distant stage disease predominated (e.g., prostate). Lastly, while a survival advantage was evident for limited stage EPSCC compared to SCLC, the advantage was less pronounced for distant stage small cell carcinoma which was associated with dismal survival across nearly all sites. The generally poor survival associated with small cell carcinoma underscores the importance of understanding disease etiology, identifying prevention/screening modalities, considering new treatment approaches, and ensuring that older patients and racially/ethnically diverse populations are included in clinical trials of new agents.

## Additional files

Additional file 1: Table S1. Stage-specific five-year relative survival of patients with small cell lung carcinoma and extrapulmonary small cell carcinoma diagnosed in SEER-13 according to gender, age, calendar year, and site, 1992-2010*.
Additional file 2: Table S2. Stage-specific five-year relative survival of patients with extrapulmonary small cell carcinoma diagnosed in SEER-13 according to site, 1992-2010*.

## Abbreviations

APC: Annual percent change
CI: Confidence interval
EPSCC: Extrapulmonary small cell carcinoma
ICD-O: International Classification of Diseases for Oncology
ICD-O-3: Third edition of ICD-O
IR: Incidence rate
IRR: Incidence rate ratio
M/F: Male-to-female
PY: Person-years
RS: Relative survival
RSR: Relative survival ratio
SCLC: Small cell lung carcinoma
SEER: Surveillance, Epidemiology and End Results
SEER-13: 13 cancer registry areas of the SEER Program
