# Supplementary material for: A population-based study of incidence and patient survival of small cell carcinoma in the United States, 1992–2010
Source: BMC Cancer. 2015 Mar 27;15:185. doi: 10.1186/s12885-015-1188-y (PMC4378011; doi:10.1186/s12885-015-1188-y)
Supplement: Additional file 1: Table S1. — Stage-specific five-year relative survival of patients with small cell lung carcinoma and extrapulmonary small cell carcinoma diagnosed in SEER-13 according to gender, age, calendar year, and site, 1992-2010*. [file 12885_2015_1188_MOESM1_ESM.docx]

| **Table S1**  **Stage-specific five-year relative survival of patients with small cell lung carcinoma and extrapulmonary small cell carcinoma diagnosed in SEER-13 according to gender, age, calendar year, and site, 1992-2010*** | | | | | | | |
| --- | --- | --- | --- | --- | --- | --- | --- |
|  | **SCLC** | | |  | **EPSCC** | | |
|  |  |  |  |  |  |  |  |
| **Stage** | **No.** | **RS (%)** | **(95% CI)** |  | **No.** | **RS (%)** | **(95% CI)** |
| **Total^†^** |  |  |  |  |  |  |  |
| Limited | 12,070 | 14.7 | (14.0, 15.4) |  | 907 | 36.2 | (32.6, 39.8) |
| Distant | 29,003 | 2.5 | (2.3, 2.7) |  | 835 | 4.7 | (3.2, 6.5) |
| **Gender and age** |  |  |  |  |  |  |  |
| Males, <60 years |  |  |  |  |  |  |  |
| Limited | 1,407 | 18.1 | (16.0, 20.2) |  | 124 | 31.7 | (23.2, 40.5) |
| Distant | 3,993 | 3.2 | (2.6, 3.8) |  | 121 | 7.0 | (3.3, 12.6) |
| Females, <60 years |  |  |  |  |  |  |  |
| Limited | 1,442 | 23.0 | (20.7, 25.3) |  | 251 | 44.4 | (37.8, 50.8) |
| Distant | 3,043 | 5.4 | (4.6, 6.3) |  | 169 | 10.2 | (6.0, 15.9) |
| Males, >60 years |  |  |  |  |  |  |  |
| Limited | 4,469 | 11.1 | (10.1, 12.2) |  | 312 | 35.7 | (29.0, 42.5) |
| Distant | 11,594 | 1.6 | (1.4, 1.9) |  | 297 | 1.5 | (0.3, 4.4) |
| Females, >60 years |  |  |  |  |  |  |  |
| Limited | 4,752 | 14.4 | (13.3, 15.5) |  | 220 | 29.4 | (22.2, 36.9) |
| Distant | 10,373 | 2.4 | (2.1, 2.8) |  | 248 | 3.1 | (1.3, 6.2) |
| **Race and age** |  |  |  |  |  |  |  |
| Whites, <60 years |  |  |  |  |  |  |  |
| Limited | 2,360 | 21.1 | (19.4, 22.9) |  | 282 | 38.6 | (32.5, 44.7) |
| Distant | 5,849 | 4.2 | (3.6, 4.7) |  | 224 | 9.0 | (5.6, 13.5) |
| Blacks, <60 years |  |  |  |  |  |  |  |
| Limited | 327 | 16.0 | (11.9, 20.5) |  | 44 | 25.1 | (13.3, 38.9) |
| Distant | 796 | 3.4 | (2.2, 4.9) |  | 36 | 10.7 | (3.2, 23.5) |
| Whites, >60 years |  |  |  |  |  |  |  |
| Limited | 7,887 | 12.9 | (12.1, 13.8) |  | 449 | 32.5 | (27.0, 38.0) |
| Distant | 18,793 | 1.9 | (1.7, 2.1) |  | 451 | 2.7 | (1.3, 5.0) |
| Blacks, >60 years |  |  |  |  |  |  |  |
| Limited | 699 | 10.7 | (8.2, 13.5) |  | 41 | 31.7 | (15.6, 49.1) |
| Distant | 1,770 | 1.9 | (1.3, 2.9) |  | 50 | 2.3 | (0.2, 10.4) |
| **Primary tumor size** |  |  |  |  |  |  |  |
| <3 cm |  |  |  |  |  |  |  |
| Limited | 2,930 | 21.7 | (20.0, 23.4) |  | 187 | 48.7 | (40.0, 56.9) |
| Distant | 4,469 | 3.6 | (3.0, 4.2) |  | 67 | 9.6 | (3.6, 19.0) |
| >3 cm - <7 cm |  |  |  |  |  |  |  |
| Limited | 3,391 | 15.3 | (14.0, 16.7) |  | 255 | 32.3 | (25.7, 39.0) |
| Distant | 6,817 | 2.6 | (2.2, 3.1) |  | 210 | 3.1 | (1.2, 6.5) |
| >7 cm |  |  |  |  |  |  |  |
| Limited | 1,071 | 10.6 | (8.7, 12.8) |  | 117 | 29.9 | (21.0, 39.4) |
| Distant | 3,079 | 3.3 | (2.6, 4.0) |  | 135 | 10.4 | (5.5, 17.1) |
| **Year of diagnosis** |  |  |  |  |  |  |  |
| 1992-2000 |  |  |  |  |  |  |  |
| Limited | 6,660 | 13.4 | (12.5, 14.3) |  | 364 | 34.5 | (29.2, 39.9) |
| Distant | 14,006 | 2.3 | (2.0, 2.5) |  | 314 | 3.8 | (2.0, 6.5) |
| 2001-2009 |  |  |  |  |  |  |  |
| Limited | 5,410 | 16.4 | (15.2, 17.5) |  | 543 | 37.3 | (32.4, 42.3) |
| Distant | 14,997 | 2.8 | (2.5, 3.1) |  | 521 | 5.2 | (3.3, 7.8) |
| Abbreviations: *CI* confidence interval, *EPSCC* extrapulmonary small cell carcinoma, *No*. number, *RS* relative survival, *SCLC* small cell lung carcinoma, *SEER-13* 13 cancer registry areas of the Surveillance, Epidemiology and End Results (SEER) Program. | | | | | | | |
| * Based on microscopically confirmed cases of small cell carcinoma diagnosed during 1992-2010 and followed through 2011. To allow a general overview of stage across primary sites, we used the SEER historic stage variable that includes localized (confined to the organ of origin), regional (direct extension to adjacent organ/tissue or regional lymph nodes), distant (discontinuous metastases), and unspecified stages. We combined localized and regional stages into the category of “limited” stage and maintained the distant stage variable as defined in the SEER Program. | | | | | | | |
| † Stage was not specified for 1,760 cases of SCLC (5-year RS (%) = 7.3, 95% CI = 6.0, 8.7), and 162 cases of EPSCC (5-year RS (%) = 17.2, 95% CI = 11.1, 24.5). | | | | | | | |
