# Supplementary material for: A population-based study of incidence and patient survival of small cell carcinoma in the United States, 1992–2010
Source: BMC Cancer. 2015 Mar 27;15:185. doi: 10.1186/s12885-015-1188-y (PMC4378011; doi:10.1186/s12885-015-1188-y)
Supplement: Additional file 2: Table S2. — Stage-specific five-year relative survival of patients with extrapulmonary small cell carcinoma diagnosed in SEER-13 according to site, 1992-2010*. [file 12885_2015_1188_MOESM2_ESM.docx]

| **Table S2 Stage-specific five-year relative survival of patients with extrapulmonary small cell carcinoma diagnosed in SEER-13 according to site, 1992-2010*** | | | |
| --- | --- | --- | --- |
|  | **No.** | **RS (%)** | **(95% CI)** |
| **Limited stage** |  |  |  |
| Uterine cervix | 164 | 42.9 | (34.6, 50.9) |
| Salivary glands | 27 | 66.6 | (35.6, 85.2) |
| Esophagus | 56 | 19.7 | (9.3, 33.0) |
| Stomach | 18 | ~ |  |
| Colon/rectum | 59 | 37.7 | (23.8, 51.5) |
| Pancreas | 15 | ~ |  |
| Larynx | 31 | 27.9 | (13.0, 44.9) |
| Female breast | 39 | 60.5 | (39.8, 76.1) |
| Ovary | 33 | 41.0 | (23.9, 57.4) |
| Prostate | 50 | 33.4 | (17.9, 49.6) |
| Urinary bladder | 256 | 34.7 | (27.7, 41.8) |
| **Distant stage** |  |  |  |
| Uterine cervix | 74 | 9.4 | (3.8, 18.0) |
| Salivary glands | 12 | ~ |  |
| Esophagus | 75 | 1.4 | (0.1, 6.7) |
| Stomach | 42 | 2.7 | (0.2, 12.2) |
| Colon/rectum | 116 | 2.2 | (0.4, 6.6) |
| Pancreas | 118 | ** |  |
| Larynx | 2 | ~ |  |
| Female breast | 14 | ~ |  |
| Ovary | 65 | 17.8 | (9.1, 28.8) |
| Prostate | 88 | 4.0 | (0.9, 11.1) |
| Urinary bladder | 87 | 2.9 | (0.6, 9.0) |
| Abbreviations: *RS* relative survival, *CI* confidence interval, *No.* number, *SEER-13* 13 cancer registry areas of the Surveillance, Epidemiology and End Results (SEER) Program, *~* relative survival not calculated for <25 cases, **** statistic could not be calculated. | | | |
| * Based on microscopically confirmed cases of small cell carcinoma diagnosed during 1992-2010 and followed through 2011. To allow a general overview of stage across primary sites, we used the SEER historic stage variable that includes localized (confined to the organ of origin), regional (direct extension to adjacent organ/tissue or regional lymph nodes), distant (discontinuous metastases), and unspecified stages. We combined localized and regional stages into the category of “limited” stage and maintained the distant stage variable as defined in SEER. | | | |
